# Integrated physiologic, proteomic, and metabolomic analyses of *Malus halliana* adaptation to saline–alkali stress

**DOI:** 10.1038/s41438-019-0172-0

**Published:** 2019-08-01

**Authors:** Xu-mei Jia, Yan-fang Zhu, Ya Hu, Rui Zhang, Li Cheng, Zu-lei Zhu, Tong Zhao, Xiayi Zhang, Yan-xiu Wang

**Affiliations:** 1 College of Horticulture, Gansu Agricultural University, 730070 Lanzhou, China; 2 Northwest Institute of Eco-Environment and Resources, Chinese Academy of Science, 730000 Lanzhou, China

**Keywords:** Metabolomics, Proteomics

## Abstract

Saline–alkali stress is a severely adverse abiotic stress limiting plant growth. *Malus halliana* Koehne is an apple rootstock that is tolerant to saline–alkali stress. To understand the molecular mechanisms underlying the tolerance of *M. halliana* to saline–alkali stress, an integrated metabolomic and proteomic approach was used to analyze the plant pathways involved in the stress response of the plant and its regulatory mechanisms. A total of 179 differentially expressed proteins (DEPs) and 140 differentially expressed metabolites (DEMs) were identified. We found that two metabolite-related enzymes (PPD and PAO) were associated with senescence and involved in porphyrin and chlorophyll metabolism; six photosynthesis proteins (PSAH2, PSAK, PSBO2, PSBP1, and PSBQ2) were significantly upregulated, especially PSBO2, and could act as regulators of photosystem II (PSII) repair. Sucrose, acting as a signaling molecule, directly mediated the accumulation of D-phenylalanine, tryptophan, and alkaloid (vindoline and ecgonine) and the expression of proteins related to aspartate and glutamate (ASP3, ASN1, NIT4, and GLN1−1). These responses play a central role in maintaining osmotic balance and removing reactive oxygen species (ROS). In addition, sucrose signaling induced flavonoid biosynthesis by activating the expression of CYP75B1 to regulate the homeostasis of ROS and promoted auxin signaling by activating the expression of T31B5_170 to enhance the resistance of *M. halliana* to saline–alkali stress. The decrease in peroxidase superfamily protein (PER) and ALDH2C4 during lignin synthesis further triggered a plant saline–alkali response. Overall, this study provides an important starting point for improving saline–alkali tolerance in *M. halliana* via genetic engineering.

## Introduction

Soil salinization–alkalization is a major abiotic stress that affects plant growth^1^. More than 831 million hectares of agricultural land is threatened by salinization and alkalinization worldwide^2^, and this problem continues to worsen. In recent years, extensive studies have focused on the mechanisms underlying the tolerance of plants to salt stress or alkali stress. However, studies on saline–alkali stress have rarely been conducted^3,4^. In northwestern China, saline–alkali stress has become a major limiting factor for apple growth and productivity^5^. The selection of saline–alkali-tolerant rootstocks is an effective strategy to minimize soil salinization and alkalinization problems^6^. *Malus halliana* is an indigenous apple species that, throughout its long evolutionary history, has already developed suitable mechanisms to adapt to saline–alkali environments. Our recent study also confirmed that *M. halliana* was more resistant to saline–alkali stress than other apple rootstocks in terms of physiological responses^7^. Consequently, exploration of the possible molecular mechanisms of how *M. halliana* responds to saline–alkali stress is urgently needed.

In plants, salt stress induces ionic stress, osmotic stress, and oxidative stress^7^. Many studies demonstrate that these stresses reduce the plant uptake of water and interfere with photosynthesis^8^, thereby affecting osmotic balance and leading to the production of reactive oxygen species (ROS)^4,5^. For instance, high-salt stress damages the photosynthetic apparatus and disturbs the expression levels of proteins related to photosynthesis, thus reducing photosynthesis and causing premature senescence^9^. Similarly, salt stress also causes dysregulation of various metabolic pathways, including signal transduction, energy metabolism, and hormone synthesis^10,11^. However, saline–alkali stress is more complex than salt stress, due to the addition of high pH stress^12^. Guo et al.^3^ have revealed that high pH disrupts ion homeostasis and accelerates the accumulation of ROS, which contributes to severe damage to cellular structures^13^. Therefore, under saline–alkali stress, plants should have more sophisticated mechanisms to re-establish osmotic and ROS homeostasis.

Numerous enzymatic scavengers play critical roles in mitigating damage induced by ROS in plants under salt stress, such as superoxide dismutase, dehydroascorbate reductase, and ascorbate peroxidase^14,15^. Zhang et al.^16^ reported that 143 proteins, which are ROS scavenging-related proteins, were induced by salinity in 24 plant species. These proteins are mainly involved in the glutathione–ascorbate cycle, catalase (CAT) pathway, PrxR/Trx pathway, and GPX pathway to alleviate ROS damage and enhance salt tolerance^17^. Moreover, in *Arabidopsis*, Hsp17.6CII (a chaperone protein) activates CAT2 activity and increases abiotic stress resistance by scavenging ROS^18^. MPK3 and MPK6 phosphorylate heat- shock factor A4A (HSFA4A) to regulate the homeostasis of ROS^19^. Intriguingly, ROS can also function as signaling molecules; plants perceive stress signals and can then transmit ROS to the cellular structures to activate defense responses that are completed by regulating related genes, protein expression, and metabolite accumulation^20^. For example, ABA pathways are important in terms of stress response signaling^17^. In apple, MdBT2, an ABA-responsive protein, interacts directly with MdbHLH93 and induces the ubiquitination and degradation of the MdbHLH93 protein, which play a significant role in delaying leaf senescence^21^. Several proteins, such as mitogen-activated protein kinases (MAPKs), PM-located protein (OSCA1), and MscS-like 8, have been to be involved in various signal transduction pathways^22–24^. In rice, MAPKs (MKK1–MPK4 cascade) play a vital role in the regulation of the expression of transcription factor genes under salt stress^25^.

Stress signals also induce the biosynthesis and accumulation of compatible osmolytes, including sugars, amino acids, and secondary metabolites. These metabolites can remove excessive ROS and lower the osmotic potential in cells^26^, which is a primary adaptive strategy in response to stress. Studies on lotus have shown that amino acids such as proline, phenylalanine, and glycine, significantly increase under salt stress^27^. A similar result was obtained by Asha et al.^11^, who reported that the accumulation of sucrose in *halophytes* resulted in the activation of antioxidant enzymes and decrease in ROS production, thus improving plant tolerance to salt stress. Importantly, recent studies have demonstrated that these metabolites also directly stimulate the related enzymes to regulate salt responses^14^. For instance, in *Arabidopsis*, free unsaturated fatty acids enhance the activity of the plasma membrane (PM) H^+^-ATPase under salt stress, which contributes to modulating protein functions and signal transduction^28^. In apple, glucose improves salt tolerance by regulating the expression of the glucose sensor MdHXK1^29^. Some of these metabolites, such as sucrose and fructose, are widely recognized as signaling molecules that trigger downstream salt-stress responses^30^. In addition, some small molecules, including proline, c-aminobutyric acid (GABA), and melatonin, mediate salt-stress signaling by stimulating the expression of related proteins^31^.

Molecular responses to stress depend on various interactions, such as those between metabolites and proteins and those between enzymes and metabolites^32^. Studies of omics, including those involving proteomics and metabolomics, offer an effective approach to obtain comprehensive insight and to connect the nodes of the molecular networks underlying the abiotic tolerance mechanisms of plants^33^. Many salt-responsive proteins and metabolites that contribute to various functions, such as photosynthesis, signal transduction, and scavenging systems of ROS, have been identified via proteomic technologies. In addition, most related differentially expressed proteins (DEPs) and metabolites are involved in metabolic processes in response to salt stress, including glycolysis (the energy metabolism pathway (EMP)), carbohydrate metabolism, the TCA cycle, and secondary metabolic pathways^11^. In recent years, significant research advances have been made in understanding plant salt-tolerance mechanisms^13,31^. However, few studies have focused on saline–alkali stress in plants. Therefore, in this study, we integrated metabolomic and proteomic analyses to identify the different proteins, metabolites, and pathways involved in the response to saline–alkali stress in plants. The objective was to gain a comprehensive understanding of how *M. halliana* regulates and responds to molecular mechanisms under saline–alkali stress, to provide an effective engineering strategy to improve plant saline–alkali tolerance.

## Results

### Selection of time points for proteomic and metabolomic analyses

**Fig. 1:**
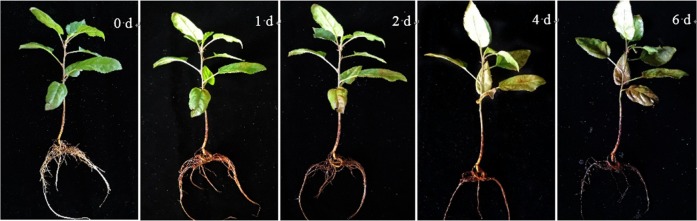
Phenotypic changes in *M. halliana* under saline–alkali stress

To determine suitable time points for our proteomic and metabolomic analyses, we observed the phenotypic changes in *M. halliana* under saline–alkali stress. The applied saline–alkali stress did not significantly alter the phenotypes of the plants for 0–2 days, as the plants showed little sign of damage. Chlorosis and dehydration in the older leaves were observed, but new leaves grew normally after 4 days of exposure to saline–alkali conditions. New leaves were chlorotic after 6 days of stress, and desiccation and crinkling of the older leaves occurred, which reflected severe stress-induced damage and even led to *M. halliana* death (Fig. 1).

### Proteomic analyses of *M. halliana* leaves in response to saline–alkali stress

The *M. halliana* leaves were assessed by iTRAQ quantitative proteomics. Quantitative analysis showed that 19,092 unique peptides corresponding to 2454 proteins were identified via LC-MS/MS determination, and the data were searched against the UniProt database. Saline–alkali stress was examined with an adjusted fold change >2 or fold change <0.5 and *p*-value <0.05 as the threshold to screen significant changes in the abundance of DEPs. We identified 179 DEPs in the leaves; among these DEPs, 74 were downregulated, and 105 were upregulated (Supplementary Table S1).

**Fig. 2:**
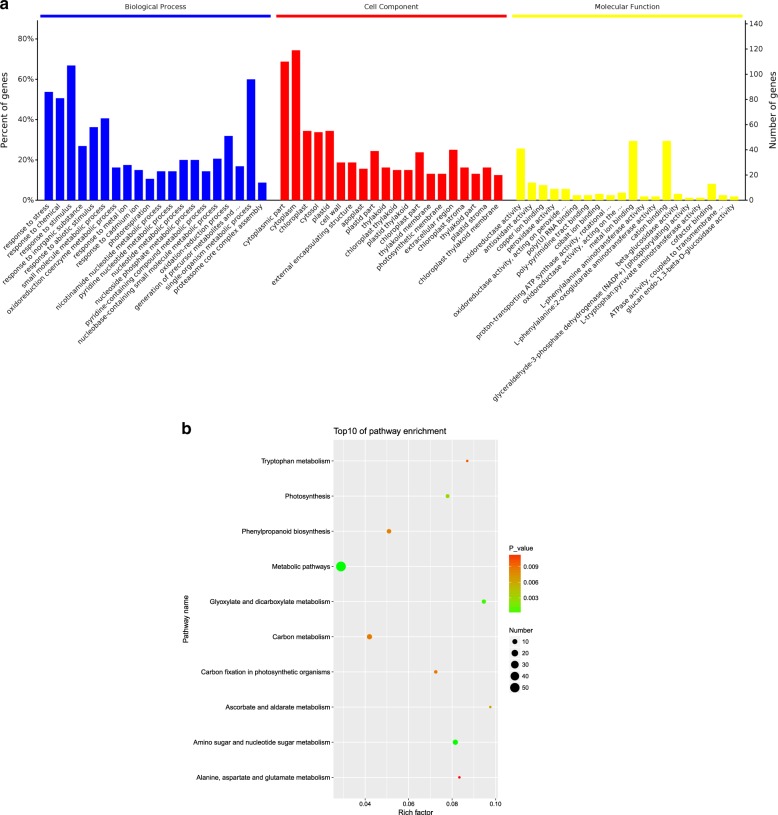
**a** GO classification of the identified proteins under saline–alkali stress. The results for the three main GO categories are summarized: BP, CC, and MF. **b** KEGG pathways of the differentially expressed proteins involved in the response to saline–alkali stress in the leaves of *M. halliana*

To investigate the functional features of these DEPs, Gene Ontology (GO) term enrichment analysis was performed using the GO and UniProt databases. The 179 DEPs were classified into three categories, including biological process (BP), cellular component (CC), and molecular function (MF) categories (Fig. 2a). In the BP category, the most abundant groups included response to a stimulus, single-organism metabolic process, and response to stress. The CC category of DEPs was significantly enriched for the cytoplasmic part, cytoplasm, and chloroplast. For the MF category, oxidoreductase activity, metal ion binding, and cation binding were the most abundant groups.

Furthermore, the proteomic results via KEGG analysis revealed that 70 pathways were enriched (Supplementary Table S2). We observed that these DEPs were involved mainly in the following metabolic pathways: amino sugar and nucleotide sugar metabolism; glyoxylate and dicarboxylate metabolism; photosynthesis; ascorbate and aldarate metabolism; phenylpropanoid biosynthesis; carbon (C) metabolism; C fixation in photosynthetic organisms; tryptophan metabolism; and alanine, aspartate, and glutamate metabolism (Fig. 2b). These results showed that proteins involved in the synthesis of metabolites also regulate various metabolic pathways in response to saline–alkali stress.

### Metabolic analyses of *M. halliana* leaves in response to saline–alkali stress

**Fig. 3:**
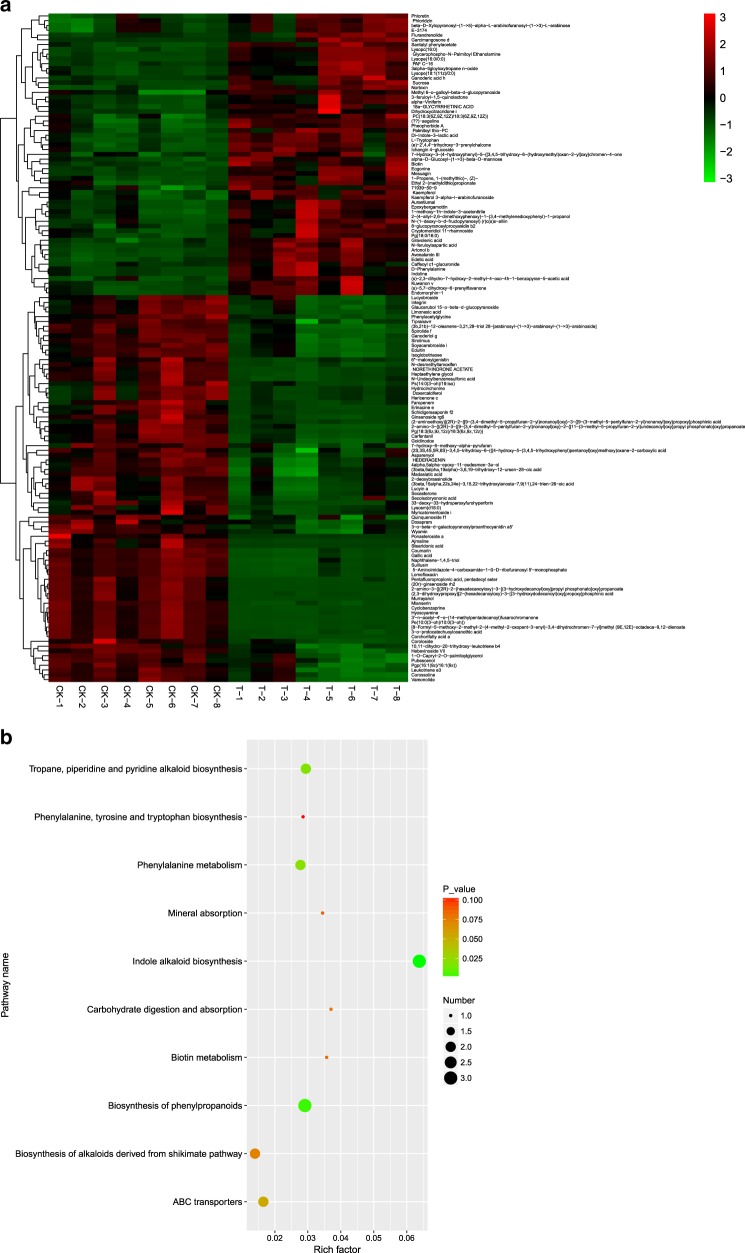
**a** Hierarchical cluster heat map of different metabolites under saline–alkali stress of *M. halliana* leaves; **b** KEGG pathways of the different metabolites involved in the response to saline–alkali stress in the leaves. “CK” represents 0 day. “T” represents 4 days. “1–8” represents repetitions

In this study, 11,135 kinds of metabolites were identified via LC–MS. Notably, 140 differentially expressed metabolites (DEMs), which included mainly sugars, glucosides, amino acids, organic acids and secondary metabolites, were selected that passed the criteria of an OPLS-DA model VIP >2.5 and had a *p*-value <0.05 in *M. halliana* leaves (Supplementary Table S3). To select marker metabolites accurately and investigate changes in the related metabolic processes, the selected DEMs were analyzed by hierarchical clustering based on the expression of significantly different metabolites in each group of samples (Fig. 3a). There was a clear separation of metabolites between the upregulated and downregulated *M. halliana* leaves. In addition, a total of 59 metabolites were upregulated, and 81 were downregulated.

All of the DEMs affected by saline–alkali stress were mapped to the KEGG database to analyze the relevant pathways. The results showed that 35 pathways were enriched with DEMs (Supplementary Table S4). The top ten most enriched items were as follows: indole alkaloid biosynthesis; phenylalanine biosynthesis; tropane, piperidine and pyridine alkaloid biosynthesis; phenylalanine metabolism; ABC transporters; shikimate pathway-derived alkaloid biosynthesis; carbohydrate digestion and absorption; biotin metabolism; mineral absorption; and phenylalanine, tyrosine and tryptophan biosynthesis (Fig. 3b). The changes in these metabolites and metabolic pathways provide important information on how *M. halliana* responds to saline–alkali stress.

### Integrated proteomic and metabolomic analyses of *M. halliana* leaves in response to saline–alkali stress

By conducting a KEGG mapping analysis based on the differences in proteins and metabolites, we found that saline–alkali stress-induced changes in the relative amounts of proteins and metabolites associated with 11 metabolic pathways, including the following: 2-oxocarboxylic acid metabolism; amino acid biosynthesis; alpha-linolenic acid metabolism; glycine, serine, and threonine metabolism; phenylalanine, tyrosine, and tryptophan biosynthesis; phenylalanine metabolism; secondary metabolite biosynthesis; tropane, piperidine, and pyridine alkaloid biosynthesis; tryptophan metabolism; phenylpropanoid biosynthesis; and metabolic pathways (Table 1).

**Table 1:** Proteins and metabolites involved in common pathways under saline–alkali stress

Pathway name	Proteomics		Metabolomics	
	Pathway ID	*P*-value	Pathway ID	*P*-value
2-Oxocarboxylic acid metabolism	map01210	0.752	ath01210	3.39E−01
Alpha-linolenic acid metabolism	map00592	0.491	ath00592	1.18E−01
Biosynthesis of amino acids	map01230	0.704	ath01230	3.23E−01
Biosynthesis of secondary metabolites	map01060	0.0147	ath01060	2.37E−01
Glycine, serine, and threonine metabolism	map00260	0.387	ath00260	1.49E−01
Metabolic pathways	map01100	1.72E−05	ath01100	5.81E−01
Phenylalanine metabolism	map00360	0.182	ath00360	2.08E−02
Phenylalanine, tyrosine, and tryptophan biosynthesis	map00400	0.285	ath00400	1.04E−01
Phenylpropanoid biosynthesis	map00940	0.00814	ath00940	1.86E−01
Tropane, piperidine, and pyridine alkaloid biosynthesis	map00960	0.143	ath00960	1.87E−02
Tryptophan metabolism	map00380	0.00992	ath00380	2.24E−01

**Fig. 4:**
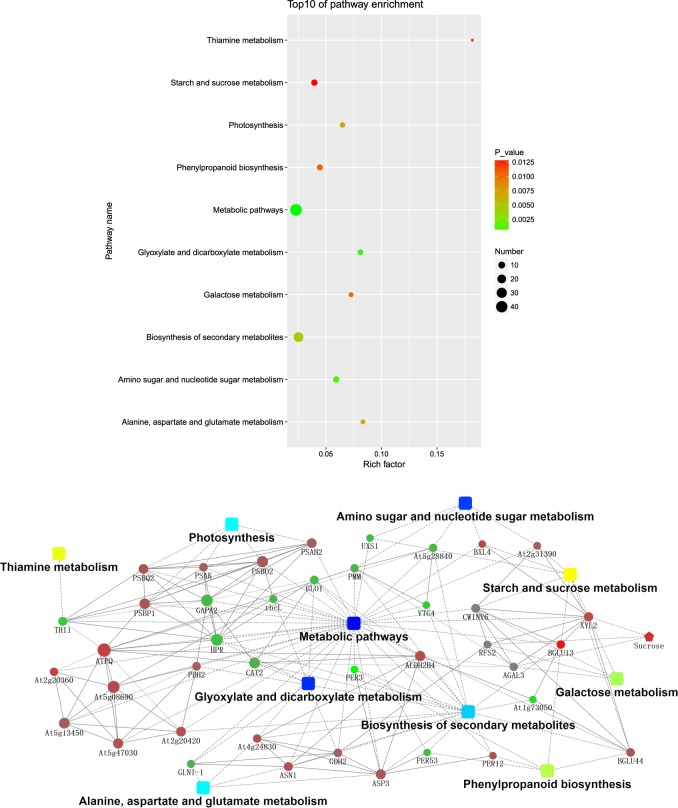
**a** KEGG pathways of the metabolite-related enzymes and DEPs involved in the response to saline–alkali stress in the leaves. **b** Network analysis of the differential proteins, metabolites, and enzymes performed using Ingenuity Pathway Analysis (IPA) software. The solid lines indicate direct interactions or regulations, while the dashed lines indicate effects mediated by additional molecules

In addition, we also performed a KEGG mapping analysis based on the differences in metabolite-related enzymes and DEPs. In total, 70 metabolic pathways exhibited changes (Supplementary Table S5), and 16 enzymes were assigned to these pathways (Supplementary Table S6). Further analysis of these metabolic pathways suggested that these proteins and enzymes were involved mainly in energy metabolism as well as in dicarboxylate metabolism; secondary metabolite biosynthesis; alanine, aspartate, and glutamate metabolism; phenylpropanoid biosynthesis; and thiamine metabolism (Fig. 4a).

Using an integrated analysis of DEPs, DEMs, and enzymes, we constructed an interactive diagram of the response of *M. halliana* to saline–alkali stress. In the network, the BGLU13 protein not only had the greatest expression fold change but also directly interacted with XYL2, CWINV6, and At1g73050, and BGLU13 was involved in both starch and sucrose metabolism and the phenylpropanoid biosynthesis pathway. In addition, metabolic pathways, glyoxylate and dicarboxylate metabolism and amino sugar and nucleotide sugar metabolism had the largest −log(*p*-value). PSBP1 can directly interact with PSBQ, PSBO2, PSAH2, and PSAK, each of which regulates the photosynthesis of *M. halliana* leaves. Only one metabolite, sucrose, which interacts with XYL2, RFSA, and AGAL3, clustered into the integrated network (Fig. 4b).

### Analysis of the comprehensive systemic metabolic pathways diagram

**Fig. 5:**
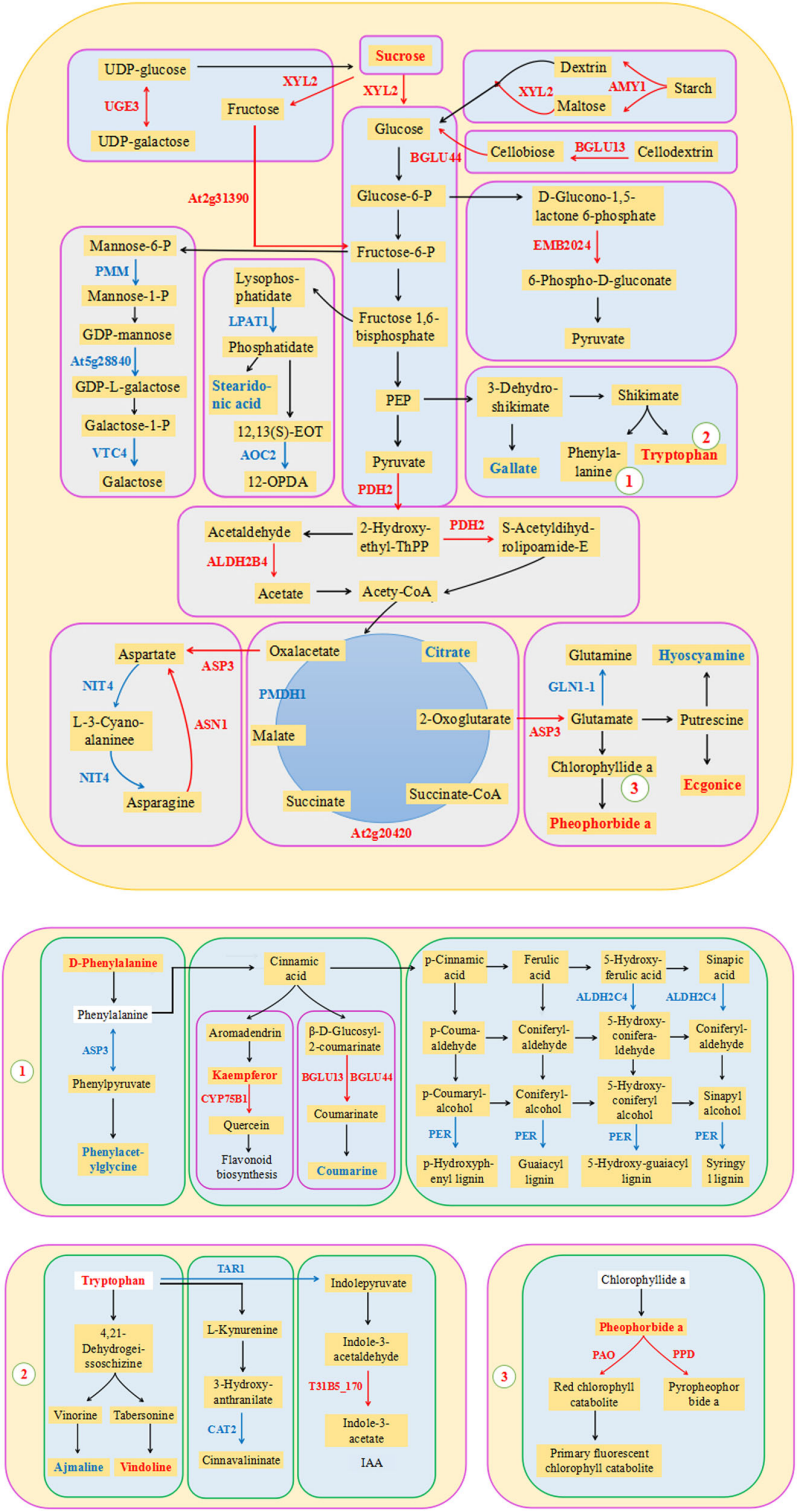
Main biological pathway responses to saline–alkali stress in *M. halliana*. Proteins (↑) and metabolites (□) were mapped to a comprehensive systemic metabolic pathways diagram by combining the KEGG pathways of the DEMs, enzymes, and proteins. The DEPs and metabolites are marked in bold; red indicates upregulation; green indicates downregulation

To explore the molecular mechanisms of *M. halliana* in regulating and adapting to saline–alkali stress further, we constructed a comprehensive systemic metabolic pathway diagram by combining the KEGG pathways of Table 1 and Fig. 5a. As shown in Fig. 5, 31 proteins and 13 metabolites were mapped to the diagram. The expression of UGE3, XYL2, At2g31390, AMY1, BGLU13, BGLU44, and sucrose, which are involved in starch and sucrose metabolism, was upregulated. Notably, the upregulation of UGE3 catalyzes the interconversion of UDP-glucose and UDP-galactose, which is the first step in the conversion of sucrose. The expression of BGLU13 increased by as much as 13.965-fold; sucrose increased by as much as 7.105-fold. In contrast, the expression of PMM, At5g28840, and VTC4, which are involved in mannose and galactose metabolism, was downregulated under saline–alkali stress (Table 2). In addition, we found high expression of PDH2 (2.246-fold), ALDH2B4 (3.503-fold), and EMB2024 (6.350-fold) in relation to the EMP and the pentose phosphate pathway (PPP). EMB2024 is involved in step 2 and synthesizes 6-phospho-d-gluconate from glucose-6-phosphate in the PPP, the former of which is a precursor to pyruvate for entry into the TCA cycle. Moreover, ALDH2B4 and PDH2 catalyze the overall conversion of pyruvate into acetyl-CoA and CO_2_ in EMP.

**Table 2:** Sugar metabolism-related proteins and metabolites

	Gene name	Protein name	UniProtKB	Fold change	*P*-value
Proteins	UGE3	Bifunctional UDP-glucose 4-epimerase and UDP-xylose 4-epimerase 3	Q8LDN8	3.498	1.806
	XYL2	Putative alpha-xylosidase 2	F4J6T7	3.845	1.943
	At2g31390	Probable fructokinase-1	Q9SID0	2.408	1.268
	AMY1	Alpha-amylase 1	Q8VZ56	4.100	2.036
	BGLU13	Beta-glucosidase 13	Q9LU02	13.965	3.804
	BGLU44	Beta-glucosidase 44	Q9LV33	2.814	1.493
	PMM	Phosphomannomutase	O80840	0.418	−1.260
	At5g28840	GDP-mannose 3,5-epimerase	Q93VR3	0.379	−1.399
	VTC4	Inositol-phosphate phosphatase	Q9M8S8	0.273	−1.874
	PDH2	Pyruvate dehydrogenase E1 component subunit beta-2	Q9C6Z3	2.246	1.168
	ALDH2B4	Aldehyde dehydrogenase family 2 member B4	Q9SU63	3.503	1.809
	EMB2024	Probable 6-phosphogluconolactonase 5	Q84WW2	6.350	2.667
	LPAT1	1-acyl-sn-glycerol-3-phosphate acyltransferase 1	Q8GXU8	0.499	−1.003
	AOC2	Allene oxide cyclase 2	Q9LS02	0.318	−1.653
Metabolites	Metabolite	Adducts	VIP	Fold-change	P-value
	Sucrose	M+Na	5.28798	7.105	0.00054
	Stearidonic acid	M+Cl	2.5174	0.029	0.00017

Significant upregulation of d-phenylalanine (5.873-fold) and downregulation of ASP3, phenylacetylglycine, and l-hyoscyamine, which are involved in phenylalanine metabolism, were detected (Table 3). In addition, the expression of BGLU13 and BGLU44, which are involved in phenylpropanoid biosynthesis, was upregulated, while the expression of coumarin and peroxidase superfamily protein (PER)12, PER53, PER52, PER17, and PER3, was downregulated in this pathway. BGLU13 and BGLU44 catalyzed the conversion of β-d-glucosyl-2-coumarinate into coumarinate and the subsequent conversion into coumarine. PER is one of the key proteins involved in lignin synthesis, and can catalyze the use of lignin monomer precursors to produce guaiacyl (G), syringyl (S), and p-hydroxyphenyl (H) lignin (Fig. 5, Table 4).

**Table 3:** Amino acid metabolism-related proteins and metabolites

	Gene name	Protein name	UniProtKB	Fold-change	*P*-value
Proteins	ASP3	Aspartate aminotransferase 3	P46644	2.471	1.305
	ASN1	Asparagine synthetase	P49078	3.228	1.690
	NIT4	Bifunctional nitrilase/nitrile hydratase	P46011	0.452	−1.147–
	GLN1–1	Glutamine synthetase cytosolic isozyme 1–1	Q56WN1	0.470	−1.088
Metabolites	Metabolite	Adducts	VIP	Fold change	*P*-value
	d-Phenylalanine	M+H	3.853	5.873	0.00060
	Phenylacetylglycine	2M−H	2.574	0.198	0.00004
	Tryptophan	M+H	2.694	7.364	1.101E−05

**Table 4:** Phenylpropanoid metabolism-related proteins and metabolites

	Gene name	Protein name	UniProtKB	Fold change	*P*-value
Proteins	CYP75B1	Flavonoid 3′-monooxygenase	Q9SD85	1.000	0.000
	BGLU13	Beta-glucosidase 13	Q9LU02	13.965	3.804
	BGLU44	Beta-glucosidase 44	Q9LV33	2.814	1.493
	ALDH2C4	Aldehyde dehydrogenase family 2 member C4	Q56YU0	0.289	−1.791
	PER3	Peroxidase superfamily protein	O23044	0.121	−3.052
	PER17		Q9SJZ2	0.274	−1.869
	PER52		Q9FLC0	0.423	−1.240
	PER53		Q42578	0.350	−1.514
Metabolites	Metabolite	Adducts	VIP	Fold change	*P*-value
	Kaempferol	M+H	3.964	1.447	0.00011
	Coumarin	M+NH_4_	2.626	0.110	6.555E−09

We further observed that the expression of kaempferol and CYP75B1, which are related to flavonoid biosynthesis, was upregulated under saline–alkali conditions (Table 4). Similarly, tryptophan (7.364-fold) and vindoline (6.220-fold), which are involved in indole alkaloid biosynthesis, significantly accumulated, but TAR1, CAT2, and ajmaline levels decreased. T31B5_170 was found to be involved in plant hormone signal transduction and was highly expressed up to 5.822-fold (Table 5). The expression of PSBO2, PSBP1, PSBQ2, PSAH2, and PSAK was significantly upregulated with respect to photosynthesis, but the expression of PSBR was downregulated; moreover, the expression of PPD, PAO, and pheophorbide a was upregulated with respect to porphyrin and Chl metabolism (Table 6).

**Table 5:** Indole and tropane, piperidine and pyridine alkaloid metabolism-related proteins, and metabolites

Proteins	Gene name	Protein name	UniProtKB	Fold change	*P*-value
	TAA1	l-tryptophan—pyruvate aminotransferase 1	Q9S7N2	0.233	−2.100
	CAT2	Catalase-2	P25819	0.461	−1.118
	T31B5_170	Auxin-responsive GH3 family protein	Q9LYU1	5.822	2.542
Metabolites	Metabolite	Adducts	VIP	Fold change	*P*-value
	Tryptophan	M+H	2.694	7.364	1.101E−05
	Ajmaline	M−H	7.007	0.027	3.619E−05
	Vindoline	M+H	2.694	6.220	0.000667
	Ecgonine	2M+K	3.171	4.316	9.578E−05
	Hyoscyamine	2M+NH_4_	2.503	0.069	1.000E−07

**Table 6:** Photosynthesis and chlorophyll metabolism-related proteins and metabolites

	Gene name	Protein name	UniProtKB	Fold change	*P*-value
Proteins	PSBR	Photosystem II subunit R	P27202	0.310	−1.688
	PSBO2	Oxygen-evolving enhancer protein 1–2	Q9S841	2.554	1.353
	PSBP1	Oxygen-evolving enhancer protein 2–1	Q42029	2.899	1.535
	PSBQ2	Oxygen-evolving enhancer protein 3–2	Q41932	3.078	1.622
	PSAH2	Photosystem I reaction center subunit VI-2	Q9SUI6	2.169	1.117
	PSAK	Photosystem I reaction center subunit psaK	Q9SUI5	2.159	1.110
	PPD	Probable pheophorbidase	O23512	1.000	0.000
	PAO	Pheophorbide a oxygenase	Q9FYC2	1.000	0.000
Metabolites	Metabolite	Adducts	VIP	Fold change	*P*-value
	Pheophorbide a	M+H	17.476	1.362	3.908E−05

### Verification of DEG analysis results by qRT-PCR

**Fig. 6:**
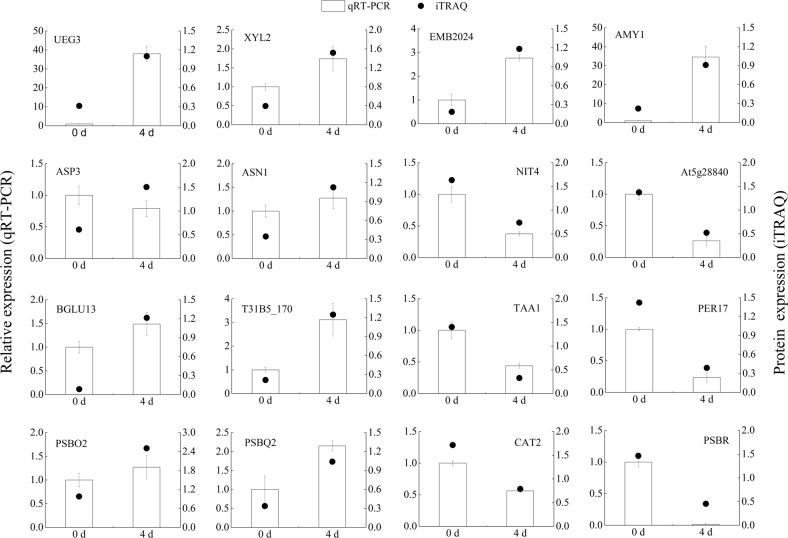
qRT-PCR confirmation of 16 proteins at two timepoints: 0 and 4 days

To further evaluate the validity of our results, 16 proteins involved in sugar metabolism, amino acid metabolism, phenylpropanoid metabolism, indole and tropane, piperidine, and pyridine alkaloid metabolism, and photosynthesis and chlorophyll metabolism were selected for quantitative real-time PCR (qRT-PCR). The results indicated that 15 genes (93.75%) showed similar trends between the qRT-PCR results and the iTRAQ analysis, but ASP3 analyzed by qRT-PCR was not consistent with iTRAQ data (Fig. 6). Interestingly, we found that ASP3 was downregulated in phenylalanine metabolism and upregulated in aspartate and glutamate synthesis under saline–alkali stress (Fig. 5). These results revealed that the iTRAQ analysis was highly reliable.

## Discussion

### Physiological indices, photosynthesis-related proteins, and Chl metabolism

Soil salinization–alkalization is a major abiotic stress that affects plant growth, production, and crop yield^34^. Saline–alkali stress leads to damage of the plant photosynthetic apparatus and even death^35^. Our physiological data showed that *P*_n_, *G*_s_, *T*_r_, Chl a, Chl b, *F*_m_, and *F*_v_/*F*_m_ in *M. halliana* decreased under saline–alkali stress, but that *F*_0_ and Y(NO) increased. The decreased *F*_v_/*F*_m_ implied that the photosystem II (PSII) reaction center induced photoinhibition, leading to a reduction in light energy use efficiency^36^. The increases in *F*_0_ and Y(NO) indicated that the photosynthetic system gradually suffered from damage^37^. This damage eventually caused an imbalance in the electron transport chain in the chloroplasts, thereby accelerating the formation of ROS and causing oxidative damage^38,39^. However, plants have evolved a number of photoprotective mechanisms. Increases in NPQ, qN, and Car are mainly thought to reflect the energy dissipation mechanism that protects the photosynthetic system by which excess energy is dissipated as heat and prevents oxidative damage^7^. In this study, the NPQ and qN in *M. halliana* did not definitively change throughout the entire stress duration, suggesting that the energy dissipation mechanism was not triggered. Therefore, we hypothesize that *M. halliana* may have triggered another stress sensing-response mechanism to avoid the production of excessive ROS in the chloroplasts.

To verify this hypothesis, we constructed a comprehensive systemic metabolic pathways diagram by combining proteomic and metabolomic approaches to explore the molecular regulatory mechanisms in response to saline–alkali stress in *M. halliana* (Fig. 5). The diagram shows that accumulated pheophorbide a was converted into both pyropheophorbide a and red Chl by PPD and PAO, respectively, which is a key step in Chl breakdown. In *Arabidopsis*, repression of ACD1 expression leads to an unusual pheophorbide a accumulation related to leaf senescence, and its accumulation ultimately results in cell death^40^. These results provide evidence that the phenotypic changes in *M. halliana* leaves may be caused by the accumulation of pheophorbide a. Chl is the main photosynthetic pigment because it is capable of harvesting, transmitting, and transforming light energy^41^. Thus, the decomposition of Chl reduced its content in the *M. halliana* leaves, thereby affecting photosynthetic efficiency and aggravating photoinhibition.

The photosystems include both photosystem I (PSI) and photosystem II (PSII)^42^. Many photosynthetic proteins have been identified in plants; for example, the LHC chlorophyll a/b-binding protein (LHC-CAB), oxygen-evolving enhancer protein (OEE), and PS II D1 protein are involved in salinity tolerance^16^. In this study, six proteins were found to be related to photosynthesis in *M. halliana* in response to saline–alkali stress, including four PSII proteins (PSBR, PSBO2, PSBP1, and PSBQ2) and two photosystem I (PSI) proteins (PSAH2 and PSAK) (Table 6). Xue et al.^43^ reported that the decrease in PSBR expression decreased the magnitude of light-induced nonphotochemical quenching (NPQ). As expected, the small change in NPQ in *M. halliana* may be a result of the downregulation of PSBR, reducing the extent of NPQ. In addition, PSBO2, PSBP1, and PsbQ2 are the oxygen-evolving enhancer (OEE) proteins of eukaryotic PSII and are required for photosynthetic water oxidation^44^. Previous studies demonstrated that PSBO2 also participates in the D1 repair cycle by regulating the turnover of the PSII D1 protein^45^, and PSBP1 and PSBQ2 are essential for the regulation of PSII assembly and/or activity^46^. These results suggested that OEE proteins play an important role in maintaining the stability and repairing the injury of the PSII reaction center to alleviate saline–alkali stress. A similar result was obtained by Zhu et al.^47^, who reported that upregulation of OEE2 repairs the injury of the PSII complex. Studies on the effects of oxidative stress on the photodamage of plants have demonstrated that ROS inhibits the repair of photodamaged PSII but does not accelerate damage to PSII^48^. Nevertheless, our study confirmed that saline–alkali stress enhanced the photoinhibition of PSII, indicating that ROS directly damages PSII. The significant upregulation of OEE proteins revealed that *M. halliana* tried to repair PSII by regulating the turnover of the D1 protein. In addition, our data showed that PSBP1 interacts with PSBQ, PSBO2, PSAH2 and PSAK (Fig. 4b); thus, we believe that PSAH2 and PSAK might be PSI-protective proteins, but their function is still unknown, which encourages additional studies to strengthen the knowledge about the changes induced by saline–alkali stress. In short, the upregulation of photosynthesis-related proteins contributed to maintaining the photosynthesis level to cope with saline–alkali stress.

### Sugar metabolism plays significant roles in many metabolic processes under saline–alkali stress

**Fig. 7:**
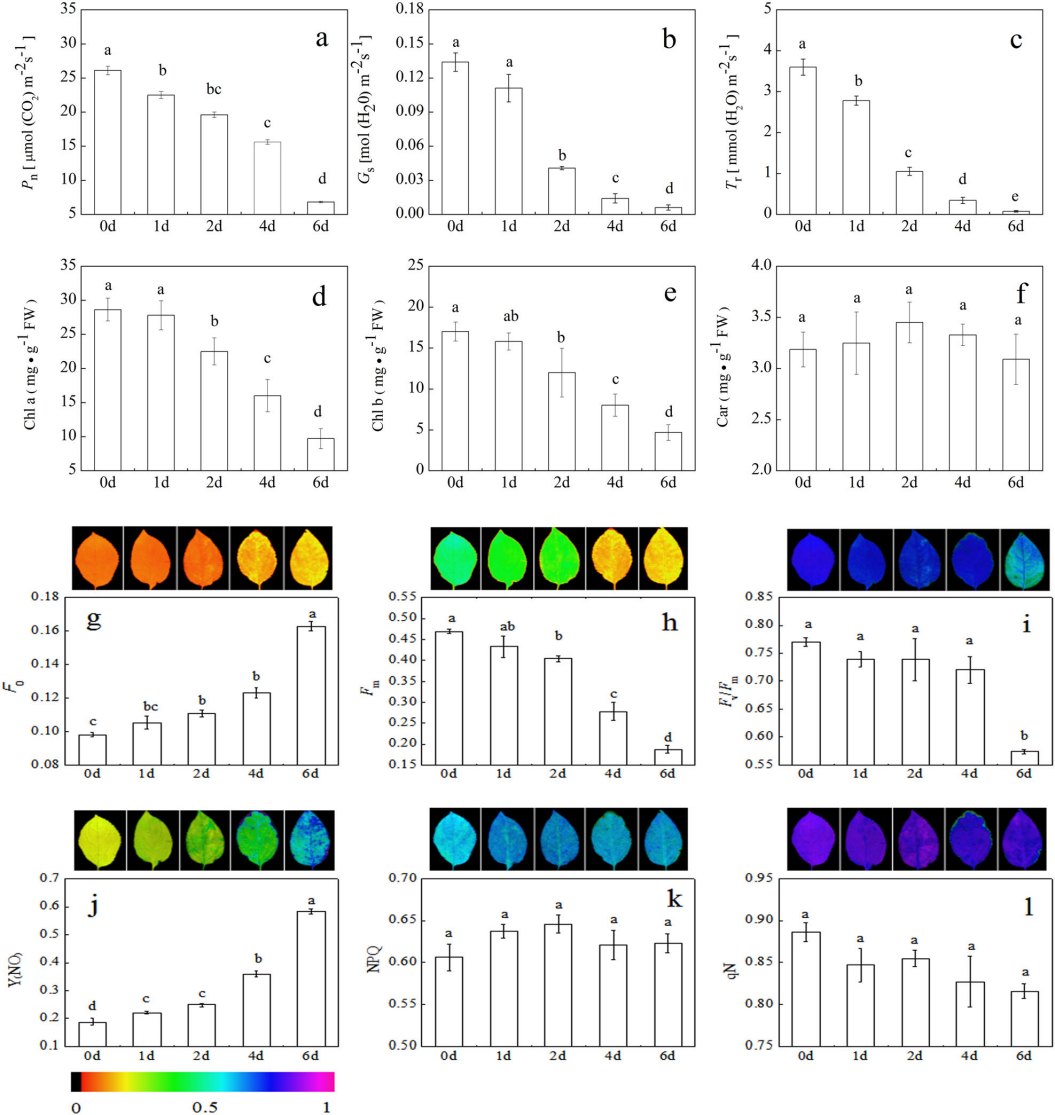
Physiological parameters of *M. halliana* under saline–alkali stress: **a** net photosynthetic rate (*P*_n_); **b** stomatal conductance (*G*_s_); **c** transpiration rate (*T*_r_); **d** Chl a content (Chl a); **e** Chl b content (Chl b); **f** carotenoid content (Car); **g** initial fluorescence (*F*_0_); **h** maximum fluorescence (*F*_m_); **i** maximal photochemical efficiency (*F*_v_/*F*_m_); **j** nonregulatory energy dissipation (Y(NO)); **k** light-induced nonphotochemical quenching (NPQ); **l** photochemical quenching coefficient (qN)

Sucrose is the primary product of photosynthesis and is widely considered an energy source for metabolism activity in plants^31^. In this study, saline–alkali stress induced a significant accumulation of sucrose and expression of UGE3 protein (Fig. 5 and Table 2). Evidence has revealed that sucrose functions as compatible osmolytes for osmotic adjustment and detoxification^31,49^, suggesting that sucrose accumulation under saline–alkali stress may be a primary strategy, by which plants protect themselves from damage by ROS and adapt to osmotic stress. In *Arabidopsis*, UGE3 plays a very critical role in the regulation of sucrose synthesis^50^. However, the photosynthesis of *M. halliana* was reduced under saline–alkali stress (Fig. 7a). These results suggested that the upregulation of UGE3 promotes the degradation of polysaccharides, thereby leading to the generation of additional sucrose to counteract saline–alkali stress. This result is similar to the result of Xu et al.^50^. In addition, sucrose also acts as a signaling molecule that regulates biotic and abiotic stress responses^30^. Sucrose-based regulation has also been observed in *Arabidopsis*, as sucrose regulates iron deficiency by promoting auxin signaling^51^. Rosa et al.^52^ confirmed that sucrose is a primary messenger that controls signaling by regulating the expression of different proteins and genes. In fact, experiments on *Camellia sinensis* have shown that sucrose induces polyphenol biosynthesis by altering the expression of transporters GST, ABC, and MATE^53^. These findings provide evidence that sucrose, which acts as a signaling molecule, might regulate many vital metabolic processes and activate other resistance pathways.

Recent studies have shown that glucose also acts as a direct and central signaling molecule in plants; this molecule was shown to improve salt tolerance by mediating the glucose sensor MdHXK1 and the vacuolar Na^+^/H^+^ transporter MdNHX1 in “Royal Gala” apple^29^. In our study, 4 significantly upregulated proteins, including XYL2, AMY1, BGLU13 and BGLU44, were involved in glucose synthesis in three different pathways (Fig. 5, Table 2). This result suggested that the pathways of glucose synthesis were enhanced under saline–alkali stress. Interestingly, in this study, glucose was not detected in *M. halliana*. Barpeled and O’Neill^54^ reported that glucose, as the energy material, was involved in various primary and secondary metabolic pathways. Studies on rice have demonstrated that EMP and PPP are important mechanisms of glucose degradation^55^. As expected, the expression of three proteins, ALDH2B4, PDH2, and EMB2024, involved in the EMP and PPP, was significantly upregulated under saline–alkali stress (Fig. 5, Table 2), which indicated that these proteins promote glucose degradation. Similar regulation was also observed by Sobhanian et al.^56^, who reported that salt stress induced the upregulation of EMP-related proteins in *Aeluropus lagopoides*. Moreover, EMP and PPP play pivotal roles in the production of both energy and the C skeletons of primary and secondary metabolites^57^. Höper et al.^58^ found that the upregulation of fructokinase-1 (FRK) contributed to glucose breakdown for energy generation to resist salt stress. These results revealed that glucose, as the main energy source, provided substrates and energy for the synthesis of other metabolites in *M. halliana*, which also explained why glucose did not accumulate under saline–alkali stress.

### Amino acid metabolism plays key roles in improving saline–alkali tolerance in *M. halliana*

Many studies have revealed that sugar metabolism provides sufficient energy to amino acid metabolism, and that intermediates from the EMP can also be utilized as precursors for the synthesis of amino acids^59^. In this study, d-phenylalanine and tryptophan, which are synthesized via the shikimic acid pathway, significantly accumulated under saline–alkali stress (Table 3). Notably, we found that the levels of all related proteins and metabolites, such as PMM, At5g28840, VTC4 and AOC2 and stearidonic acid, were downregulated in galactose metabolism and alpha-linolenic acid metabolism (Fig. 5, Table 2), which indicated that the two pathways were inhibited by saline–alkali stress in *M. halliana*. This phenomenon might be caused by a decrease in the consumption of galactose and stearidonic acid synthesis and an increase in energy supplies for amino acid metabolism. In higher plants, amino acids accumulate in response to various stresses and have multiple functions in plant growth^60^. However, our results showed no altered protein levels (Fig. 5). Similar results were obtained in alfalfa, in which l-tyrosine and phenylalanine were upregulated but in which upregulated levels of related proteins were not detected. Fan et al.^32^ inferred that this upregulation may have resulted from protein hydrolysis. In potato tubers, it has also been postulated that amino acid accumulation is a mechanism to compensate for cellular osmolarity caused by reductions in sucrose concentration^61^. Sugar metabolism analyses have revealed that sucrose, as a signaling molecule, can transmit stress signals to other pathways and regulate the expression of related proteins and metabolites to respond to adversity^52^. We therefore speculate that the accumulation of both d-phenylalanine and tryptophan may be induced by sucrose signals in response to saline–alkali stress.

In this study, d-phenylalanine is reversibly converted into phenylalanine, leading to the production of phenylacetylglycine after a series of reactions in phenylalanine metabolism. The downregulation of ASP3 results in a decrease in phenylacetylglycine, suggesting that *M. halliana* limits phenylalanine metabolism to resist saline–alkali conditions (Fig. 5, Table 3). In contrast, the upregulation of ASP3, which is involved in aspartate and glutamate synthesis, indicates that ASP3 might be a signaling-related protein. The upregulated expression of ASP3 can initiate amino acid defense reactions to resist saline–alkali stress^62^. In addition, the accumulation of amino acids (proline, serine, phenylalanine, and threonine as osmolytes) can improve plant tolerance to salt stress by mediating the removal of ROS and damage control and repair^13,27^. In addition, these amino acids serve as precursors for a large array of secondary metabolites, including pigments, alkaloids, hormones, and cell wall components^63^.

### Sucrose signaling regulates ROS homeostasis by inducing phenylpropanoid biosynthesis pathway and flavonoid synthesis

Considerable evidence exists that phenylalanine, which is required for the biosynthesis of flavonoids and lignin, is an important precursor of phenylpropanoids^57^. In this study, kaempferol was significantly upregulated and was subsequently converted into quercetin by upregulated CYP75B1, thus enhancing flavonoid biosynthesis (Fig. 5, Table 4). Similarly, GmMYB173 has been reported to improve soybean salt tolerance by regulating flavonoid biosynthesis^64^. Quercetin and kaempferol are well-known flavonol glycosides that play a positive role in scavenging excess ROS^65^. Notably, Dubos et al.^66^ found that sucrose signaling induced flavonoid biosynthesis by activating the expression of MYBL2 in *Arabidopsis*. This finding might provide strong evidence that sucrose signaling regulates flavonoid synthesis to control the homeostasis of ROS under saline–alkali stress.

Furthermore, our results showed that most of the proteins and metabolites were downregulated in the phenylpropanoid biosynthesis pathway, especially the proteins involved in lignin synthesis, such as PER3, PER17, PER12, PER52, PER53 (PER, peroxidase superfamily protein), and ALDH2C4 (Fig. 5, Table 4). This finding suggested that lignin synthesis was inhibited by saline–alkali stress in *M. halliana*. Lignin provides mechanical strength to plant secondary cell walls^67^, which not only protect cells from abiotic stress but also are serve as the structures that first perceive and respond to environmental stress^68^. In alfalfa, antisense downregulation of the enzyme hydroxycinnamoyl-CoA: shikimate hydroxycinnamoyl transferase (HCT) led to decreased lignin content. Gallego-Giraldo et al.^69^ reported that the downregulation of lignin biosynthesis might be a defense mechanism against abiotic stress. These results demonstrated that cell wall damage and altered components in response to salt stress are possible salt-sensing mechanisms that would further trigger plant salt responses^31^. In addition, studies in trees have described that phenylpropanoids represent a significant pathway for C and energy flow during lignin biosynthesis^70^. Hence, a decrease in phenylpropanoid biosynthesis might conserve C skeletons and energy for flavonoid biosynthesis.

In this study, BGLU13 and BGLU44 were upregulated in the phenylpropanoid biosynthesis pathway, which is involved in the synthesis of coumarine. However, coumarine was downregulated by saline–alkali stress (Fig. 5, Table 4). This result indicated that BGLU13 and BGLU44 might be defense-related proteins and that their upregulation might protect plants against saline–alkali stress. Previous studies have demonstrated that defense proteins play a pivotal role in repairing oxidative damage^71^. These results revealed that phenylpropanoid metabolism is a key defense signaling pathway.

### Sucrose signaling regulates ROS homeostasis by inducing alkaloid metabolism and auxin synthesis

Alkaloids, which are secondary metabolites that play a role in defense against stresses, such as salinity^72^ and water^73^ stress, are synthesized from tryptophan and glutamate. Studies on *Catharanthus roseus* have demonstrated that the upregulated expression of *sgd*, ORCA3, and *t16h* may contribute to the observed increases in ajmalicine, vindoline, and catharanthine following binary stress^74^. In the present study, vindoline was highly upregulated, but no related proteins were identified in the indole alkaloid pathways (Fig. 5, Table 5), indicating that the accumulation of vindoline may be a defense response to saline–alkali stress. Similar regulation was also observed by Li et al.^75^ who reported that vindoline accumulation increased the scavenging capacity of ROS, thereby providing protection against oxidative injury and reducing metabolic disturbance in plants. However, in our study, ajmaline levels were downregulated, and no related proteins were found in the pathway (Fig. 5). One possible explanation is that tryptophan is the first step of indole alkaloid synthesis and that its accumulation provides skeletons for vindoline and ajmaline^76^. The reduced ajmaline synthesis may provide energy for vindoline synthesis in *M. halliana*. Our results showed that the response mechanism of ecgonine and hyoscyamine was typically consistent with that of vindoline and ajmaline. As a result, we believe that the changes in alkaloid levels may be regulated by sucrose signaling, and vindoline and ecgonine, as nonenzymatic scavengers, can mitigate damage induced by ROS to increase saline–alkali tolerance in *M. halliana*.

In this study, sucrose signaling also mediated auxin biosynthesis in *M. halliana*. Auxins, of which indole-3-acetic acid (IAA) is the main active form in higher plants, regulate many aspects of plant growth and development, including seed germination, root architecture, and leaf formation^77,78^. However, several recent studies have highlighted that auxin also regulates plant responses to stresses, such as drought and salinity tolerance^79^. We observed that the expression of T31B5_170, an auxin-responsive GH3 family protein, was significantly upregulated under saline–alkali stress and was involved in IAA synthesis (Fig. 5, Table 5). GH3 family proteins play critical roles in the regulation of auxin homeostasis^80^. In *Arabidopsis*, several members of the GH3 protein family activate IAA by adenylation^81^. Recent studies have demonstrated that auxin homeostasis under alkaline stress contributes to the adaptation of citrus to alkaline stress. These results indicated that T31B5_170 may induce the auxin signaling pathway that enhanced the resistance of *M. halliana* to saline–alkali stress.

## Conclusions

**Fig. 8:**
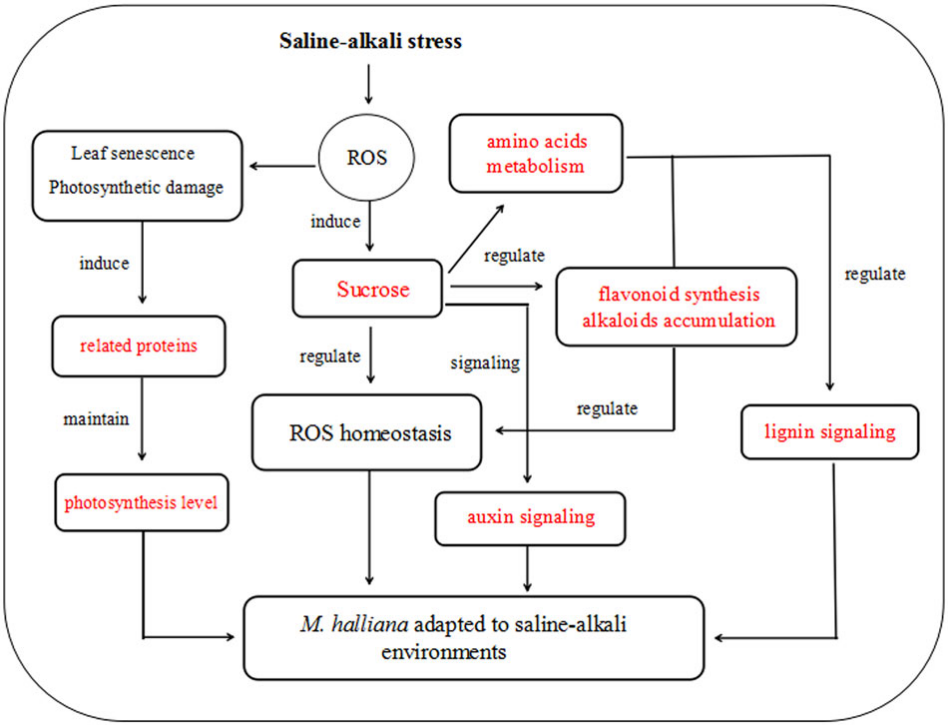
Signal transduction of ROS during the plant response to saline–alkali stress

In summary, saline–alkali stress induced the accumulation of ROS. To adapt to stress, *M. halliana* relied on signals and pathways to re-establish the homeostasis of ROS. The upregulated expression of photosynthesis-related proteins enhanced the capacity of photosynthesis in *M. halliana*, *which* contributed to maintaining the photosynthesis level to cope with saline–alkali stress. Sucrose acted as a signaling molecule and directly mediated amino acid metabolism; phenylpropanoid metabolism; and indole, tropane, piperidine, and pyridine alkaloid metabolism. These pathways play a central role in maintaining osmotic balance and removing ROS. In addition, sucrose signaling induced flavonoid biosynthesis by activating the expression of CYP75B1 to regulate the homeostasis of ROS and promoted auxin signaling by activating the expression of T31B5_170 to enhance the resistance of *M. halliana* to saline–alkali stress. The decrease in PER and ALDH2C4 during lignin synthesis further triggered the plant saline–alkali response (Fig. 8).

This is the first study that has used an integrated approach to determine the possible molecular mechanisms of how *M. halliana* adapts to saline–alkali environments. Moreover, this study provides the basis for an improved understanding of saline–alkali tolerance responses in apples and provides an important starting point for future analyses. Thus, identifying specific saline–alkali stress sensors/receptors and exploring their underlying mechanisms via genetic engineering remain exciting challenges.

## Materials and methods

### Plant material and stress treatments

The experiment was carried out at Gansu Agricultural University (Gansu Province, China) in May 2018. *M. halliana* seeds were sterilized with 0.2% KMnO_4_ for 30 min and then washed with running water for 12 h. Subsequently, seeds in sand were germinated at 4 °C for 35 days and shown in 16.8-cm-diameter plastic pots that contained 2.5 kg of nutrient soil. Healthy seedlings with eight true leaves were selected and transferred to foam boxes containing Hoagland’s solution (pH = 6.8 ± 0.2)^7^. Five seedlings were grown in each box. The nutrient solution was changed every 3 days, and oxygen was continuously ventilated by an electric ventilation pump during the cultivation period. After 12 days, ten boxes with uniform seedlings were selected and subjected to saline–alkali stress, which involved treatment with Hoagland’s solution supplemented with 100 mM NaCl + NaHCO_3_ (1:1 M ratio of NaCl:NaHCO_3_) (pH = 8.2). To avoid any salt-shock reaction, an increase of 25 mM concentration per day was applied at the beginning of the stress. All physiological parameters were measured after reaching the 100 mM concentration.

### Determination of photosynthetic parameters

The photosynthetic characteristics of functional leaves at 0, 1, 2, and 4 days were measured from 9:00 to 11:00 a.m. using an LI-6400XT portable open-flow gas-exchange system (Li-COR Biosciences, Lincoln, USA). After 30 min of the darkness treatment, we used an Imaging-Pam Chl fluorimeter and Imaging WinGegE software (Walz, Effeltrich, Germany) to determine the chlorophyll (Chl) fluorescence parameters of leaves. The Chl content was extracted with 10 mL of acetone for 48 h in darkness and measured at 440, 645, and 663 nm spectrophotometrically. The calculations used the methods of Lichtenthaler^82^.

### iTRAQ analysis methods

#### Protein extraction, protein digestion, and iTRAQ labeling

Proteins were extracted from three biological replicates per treatment, and 0.1 g of frozen leaf tissue was placed into a cold mortar. A mortar and pestle were used to grind tissue into a fine powder, and 1 mL of phenol extraction buffer was added to incubate at room temperature for 10 min. Then, 1 mL of phenol saturated was added, and the mixture was shaken for 40 min at 4 °C. Tubes were centrifuged at 5000 *g* for 15 min at 4 °C, and the upper phenolic phase was collected. Subsequently, cold 0.1 M ammonium acetate–methanol solution was added at −20 °C using five volumes of the collected phenolic phase. The sediment was collected after centrifugation at 12,000 *g* for 10 min at 4 °C. Then, the sediment was collected, repeating this step one more time, and dried at room temperature for 2 min. Subsequently, the sediment was resuspended in 300 μL of lysate solution for 3 h. Finally, the supernatant was the extracted protein solution after centrifugation of mixtures at 12,000 *g* for 10 min at room temperature. Measurements of protein were performed by the Bradford method^83^. iTRAQ labeling was performed according to the FASP method^84^.

#### Mass spectrometry analysis

All samples were analyzed by a Triple TOF 5600 mass spectrometer (SCIEX, USA). The flow rate of the Eksigent nanoLC-1D plus system (SCIEX, USA) was 300 nL/min, and the linear gradient was 90 min (from 5 to 85% B over 67 min; mobile phase A = 2% ACN/0.1% FA and B = 95% ACN/0.1% FA). A rolling collision energy voltage was used for CID fragmentation for MS/MS spectra acquisitions. Mass was dynamically excluded for 22 s.

#### Bioinformatics analysis

To analyze the functional characteristics of the selected DEPs, GO functional annotations and enrichment analysis of the proteins were performed by using the cloud platform of OmicsBean. For this analysis, the online Kyoto Encyclopedia of Genes and Genomes (KEGG, http://www.kegg.jp/) was used to classify the identified proteins. In addition, noncommercial databases, including metabolite pathways, were searched on KEGG (http://www.genome.jp/KEGG/pathway.html).

### LC/MS untargeted metabolomics analysis methods

#### Sample preparation

A total of 80 mg of accurately weighed sample was transferred to a 1.5-mL Eppendorf tube. Then, 1 mL of a mixture of methanol and water (7/3, vol/vol) was added to each tube, and 20 μL of 2-chloro-l-phenylalanine (0.3 mg/mL) was dissolved in methanol to serve as the internal standard. Samples were ground at 60 Hz for 2 min, ultrasonicated at room temperature for 30 min after vortexing, and then placed at 4 °C for 10 min. After centrifugation at 13,000 rpm at 4 °C for 15 min, the supernatants (200 μL) from each tube were collected.

#### LC/MS analysis

LC/MS analysis was performed using an Acquity UHPLC system (Waters Corporation, Milford, USA) coupled with an AB Sciex Triple TOF 5600 System (AB Sciex, Framingham, MA). Data acquisition was conducted in full-scan mode (the *m*/*z* ranged from 70 to 1000) in combination with information-dependent acquisition mode. The parameters were set as follows: ion spray voltage, 5500 V (+) and 4500 V (−); ion source temperature, 550 °C (+) and 550 °C (−); collision energy, 10 eV (+) and −10 eV (−); curtain gas of 35 PSI; interface heater temperature, 550 °C (+) and 600 °C (−).

#### Data preprocessing and statistical data analysis

The raw data were converted to common data format (mzML) files using the software program MSconverter, and metabolomic data were obtained using the software XCMS 1.50.1 version. The positive and negative data were combined to obtain a combined data set that was imported into the SIMCA software package (version 14.0, Umetrics, Umeå, Sweden). All samples were tested to visualize the metabolic alterations by principal component analysis and (orthogonal) partial least-squares-discriminant analysis (O)PLS-DA. Variable importance in the projection (VIP) ranks the overall contribution of each variable to the OPLS-DA model, and those variables with VIP >1 were considered relevant for group discrimination. Reference material databases built by Dalian Institute of Chemical Physics, Chinese Academy of Sciences, and Dalian ChemData Solution Information Technology Co., Ltd., HMDB and METLIN, were used. In addition, metabolite pathways were searched on noncommercial databases (KEGG, http://www.genome.jp/KEGG/pathway.html).

#### Quantitative real-time PCR

The expression of 16 proteins was measured by performing real-time quantitative PCR (qRT-PCR). cDNA was synthesized from total RNA using the PrimeScript™ RT reagent Kit with gDNA Eraser (Perfect Real Time) (TaKaRa, Dalian, China) according to the manufacturer’s instructions. GAPDH was used as an internal control gene and qRT-PCR method based on Pan et al.^85^. The qRT-PCR analysis of each sample was performed in triplicate. Primers for the quantitative real-time PCR are listed in Table 7.

**Table 7:** Primer list for the qRT-PCR analysis of *M. halliana* genes

Accession	Gene	Forward primer (5′-3′)	Reverse primer (5′-3′)
MD04G1225400	UGE3	CATGCGGAAGGTGCCTCTTGG	AATCCTCCGTTGCTGCACTGATC
MD13G1042700	XYL2	GAGGCTTACGCACACTCTGTTCTG	GCCGATGAACGCCGTGTACTG
MD01G1226300	EMB2024	TCGGCGTAGGCAGAGATGGC	ACAGGTCGGCGGTGTACTTCG
MD08G1101700	AMY1	GCTGCCGAGGCTAACCTGTATATG	ACTGGTGGCAACGTGGAAGTTC
MD04G1224200	At5g28840	GTCAGACTTCCGTGAGCCAGTG	TGATCAGCGTGTTGTCCGAGTTAC
MD15G1096900	ASP3	ACGAGTAGTGATCGGAGGCTGAG	GCGAGGCAGAGATGGAGTTGTTG
MD03G1066000	ASN1	AGCATCGTGGACCTGACTGGAG	TTCGGAACTTGTGATTCGGCAGAC
MD03G1150700	NIT4	CTCCTTCTTCAGCCGCATACACAG	TAGAAGACGGTGGAGGCTTGGAC
MD03G1068200	BGLU13	CGGCTGCTTCAAGCTGGCTAG	AGATAGTCGAGGTGGTGGTAGTGG
MD15G1321300	PER17	AACAGCAAGCCAAGAAGCCTCAG	GGCCACTGCCAGATTGGTTGTAC
MD16G1149300	T31B5_170	CCACCGCCATTGTCTTCCTCATC	GAGCCGCCGTGGTGATGTTG
MD06G1009000	CAT2	GATCCTGTTCGCCATGCTGAGAC	CATCGGTGGAGGAATCGCTCTTG
MD10G1035800	TAA1	GGCGTAGAGGTAGCGTGTATGC	TTCCTTGACCAACTGGCTTAGTCG
MD15G1272500	PSBO2	CCTCTGGCAAGCCTGACAACTTC	CCAACTCCTCCTCGTCTCCTCTG
MD10G1311700	PSBQ2	GCTTGGTCTCGTCGCAGTTGG	CTTCAACGGCAGGTCAAGGTCTC
MD10G1198300	PSBR	TCATTCAAGGTGCAAGCCAGTGG	CCAGAGGCATCAACACCGTTCC

### Statistical analysis

Statistical differences were tested by analyses of variance, and the mean differences were compared using Duncan’s test (*P* < 0.05). Statistical analyses were analyzed using SPSS (version 22.0, IBM, Armonk, NY, USA), and figures were made using Origin 9.1 software (OriginLab, Hampton, MA, USA).

## Supplementary information

Supplementary Table S6

Supplementary Table S1

Supplementary Table S2

Supplementary Table S3

Supplementary Table S4

Supplementary Table S5
